# Tri-2-Hydroxyarachidonein Induces Cytocidal Autophagy in Pancreatic Ductal Adenocarcinoma Cancer Cell Models

**DOI:** 10.3389/fphys.2021.782525

**Published:** 2022-01-20

**Authors:** Javier Fernández-Díaz, Roberto Beteta-Göbel, Manuel Torres, Joan Cabot, Paula Fernández-García, Victoria Lladó, Pablo V. Escribá, Xavier Busquets

**Affiliations:** ^1^Laboratory of Molecular Cell Biomedicine, Department of Biology, University of the Balearic Islands, Palma de Mallorca, Spain; ^2^Laminar Pharmaceuticals, Department of R&D, Palma de Mallorca, Spain

**Keywords:** synthetic lipid, cell death, pancreas, signal transduction, oncology, autophagy, cancer

## Abstract

Cell proliferation in pancreatic cancer is determined by a complex network of signaling pathways. Despite the extensive understanding of these protein-mediated signaling processes, there are no significant drug discoveries that could considerably improve a patient’s survival. However, the recent understanding of lipid-mediated signaling gives a new perspective on the control of the physiological state of pancreatic cells. Lipid signaling plays a major role in the induction of cytocidal autophagy and can be exploited using synthetic lipids to induce cell death in pancreatic cancer cells. In this work, we studied the activity of a synthetic lipid, tri-2-hydroxyarachidonein (TGM4), which is a triacylglycerol mimetic that contains three acyl moieties with four double bonds each, on cellular and *in vivo* models of pancreatic cancer. We demonstrated that TGM4 inhibited proliferation of Mia-PaCa-2 (human pancreatic carcinoma) and PANC-1 (human pancreatic carcinoma of ductal cells) in *in vitro* models and in an *in vivo* xenograft model of Mia-PaCa-2 cells. *In vitro* studies demonstrated that TGM4 induced cell growth inhibition paralleled with an increased expression of PARP and CHOP proteins together with the presence of sub-G_0_ cell cycle events, indicating cell death. This cytocidal effect was associated with elevated ER stress or autophagy markers such as BIP, LC3B, and DHFR. In addition, TGM4 activated peroxisome proliferator-activated receptor gamma (PPAR-γ), which induced elevated levels of p-AKT and downregulation of p-c-Jun. We conclude that TGM4 induced pancreatic cell death by activation of cytocidal autophagy. This work highlights the importance of lipid signaling in cancer and the use of synthetic lipid structures as novel and potential approaches to treat pancreatic cancer and other neoplasias.

## Introduction

The pancreatic ductal adenocarcinoma (PDAC) is the fourth leading cause of cancer death in the United States and the sixth in Europe (Jemal et al., 2009). PDAC is the most common pancreatic neoplasm, which comprises approximately 90% of all pancreatic malignancies (Ferlay et al., 2010). Advanced age together with smoking, diabetes, obesity, or pancreatitis are contemplated risk factors (Everhart and Wright, 1995; Gapstur et al., 2000; Michaud et al., 2001; Huxley et al., 2005). Despite recent advances in chemotherapy including gentamicin in combination with other agents, adjuvants, and immunotherapy, the survival for pancreatic cancer remains grim (Roth et al., 2020).

Cell proliferation in pancreatic cancer is determined by an enormous complex network of signaling pathways (Polireddy and Chen, 2016). K-Ras mutations are often contemplated as the main initiating event, latter followed by P16 mutations, P53, and SMAD4 loss (Jones et al., 2008). Despite the extensive familiarity of these protein-mediated signaling networks, there are no significant advances in treatment strategies. However, the recent understanding of lipid-mediated signaling gains importance in understanding the control of the physiological state of pancreatic cells (Swierczynski et al., 2014).

In this context, cancer cells to supply the requirement of membrane phospholipids due to their altered proliferative state are characterized by an activation of the fatty acid biosynthetic pathways (Swinnen et al., 2000; Kuhajda, 2006). For example, the activity of AMPK in PDAC cells is lower than in normal cells, leading to a rise in the activity of acetyl-CoA carboxylase, a key factor in lipogenesis (Adachi et al., 2011). The phosphorylation by AMPK, causing acetyl-CoA carboxylase inhibition, is one of the central stages of lipogenesis (Saha and Ruderman, 2003) together with the increased expression of/fatty acid synthase in human pancreatic cancer (Witkiewicz et al., 2008; Yang et al., 2011). The elevated levels of fatty acid synthase, both in tumor cells and in serum, are even associated with poor prognosis (Walter et al., 2009).

Interestingly, several saturated and unsaturated fatty acids modulate autophagy (Brenner et al., 2013; Niso-Santano et al., 2015). Autophagy is a cellular process that controls the recycling of macromolecules and cytoplasmic structures through phagosomes (Mizushima et al., 2008; Martinet et al., 2009). Autophagy has also been associated with the induction of non-apoptotic cell death (Miller et al., 2019). The accumulation of misfolded proteins in the endothelium reticulum results in the upregulation of the unfolded protein response (UPR) pathway and the expression of autophagy-related genes (Ogata et al., 2006; Kouroku et al., 2007). UPR and autophagy can either cause cytoprotective or cytocidal effects (Kondo et al., 2005; Moenner et al., 2007; Yonekawa and Thorburn, 2013).

Nowadays, it is well established that lipid signaling plays a major role in the induction of autophagy (Wang and Nie, 2015; Soto-Avellaneda and Morrison, 2020). The cytocidal effects of autophagy can be exploited using synthetic fatty acids to induce cell death in cancer cells and represent a promising field in cancer therapy investigation (Marcilla-Etxenike et al., 2012). In fact, autophagy is upregulated in PDAC and is regarded as a potential therapeutic target in PDAC and other cancers (Piffoux et al., 2021). Previously, we demonstrated that sustained activation of autophagy with synthetic fatty acids triggers cancer cell death (Marcilla-Etxenike et al., 2012; Teres et al., 2012; Beteta-Göbel et al., 2021). In this work, we studied the activity of a synthetic triacylglycerol, TGM4, on cellular and *in vivo* models of pancreatic cancer. We demonstrated that TGM4 induces pancreatic cell death by the activation of autophagy. This work highlights the importance of lipid signaling in cancer and the use of synthetic lipid structures as novel and potential approaches to treat pancreatic cancer and other neoplasias.

## Materials and Methods

### Tri-2-Hydroxyarachidonein

**FIGURE 1:**
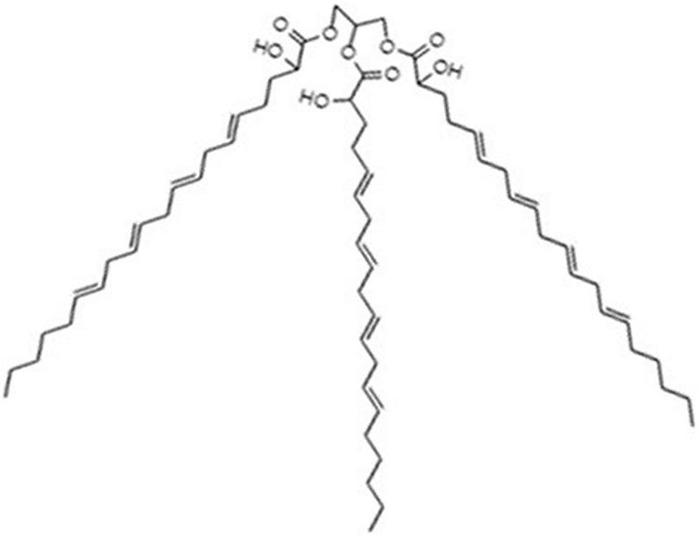
Molecular structure of TGM4. This compound is a triacylglycerol mimetic containing three 2-hydroxyarachidonyl moieties.

The compound, tri-2-hydroxy-eicosatetra (5E,8E,11E,14E) enoine (also tri-2-hydroxyarachidonein or TGM4; Figure 1), was kindly provided by Laminar Pharmaceuticals (Palma, Spain).

### Cell Culture

Human pancreatic carcinoma (Mia-PaCa-2) and human pancreatic carcinoma of ductal cells (PANC-1) were obtained from American Type Culture Collection (ATCC). Both cell lines were maintained and grown in 75-cm^2^ flasks with Dulbecco’s modified Eagle’s medium (DMEM) with phenol red including 10% of fetal bovine serum (FBS), 100 U/ml of penicillin, and 100 μg/ml of streptomycin supplemented with D-glucose (4.5 g/L), L-glutamine (4 mM), and sodium pyruvate (1 mM). They were incubated in HEPA-filtered cell incubator (Memmert GmbH Co., United Kingdom) at 37°C with 95% humidified air and 5% CO2. Cell culture experiments were carried on a laminar vertical flow cabinet (Telstar S.A., Terrasa, Spain).

### Cell Proliferation or Cytotoxicity Studies

For cytotoxicity experiments, Mia-PaCa-2 and PANC-1 cell lines were seeded at a density of 3 × 10^3^ cells/well into 96-well plates and incubated at 37°C for 24 h in a CO_2_ incubator. After 24 h, the medium was changed and replaced with a new medium containing TGM4. To dissolve the TGM4, a stock solution of 100 mM of TGM4 was prepared in DMSO. Then, the stock solution was dissolved in DMEM medium to obtain the desired concentration, never surpassing the 0.5% of final DMSO in the medium.

The cytotoxic effects were measured using the cell proliferation XTT kit (Roche Diagnostics, S.L. Applied Science, Barcelona, Spain). After incubation of cells with TGM4, the medium was replaced with DMED without phenol red mixed with the XTT reagents, following the instructions of the manufacturer. Then, the cells were incubated at 37°C until the color compound was formed. The absorbance was measured at a wavelength of 495 nm in a plate reader (FLUOStar OMEGA, BMG LABTECH, Germany). For statistical confidence, four independent experiments were performed. The IC_50_ (inhibitory concentration 50) was calculated with GraphPad Prism version 5.

### Cell Cycle Studies

Human pancreatic carcinoma of ductal cells were seeded at a density of 250,000 cells into 6-cm-diameter plates and incubated at 37°C for 24 h in CO_2_ incubator. After 24 h, the medium was changed and replaced with new medium containing TGM4 (20, 30, or 40 μM). The cells were incubated for 6, 12, 24, or 48 h.

After incubation, the cells were recovered by centrifugation at 1,000 × *g* for 5 min in 5-ml cytometry tubes and suspended in complete media. Then, cells were detached with EDTA-trypsin and centrifuged at 1,000 × *g* for 5 min. The supernatant was discarded and all the cells were stored in the tube.

To fix the cells, cold 70% ethanol was added to the tube while vortexing. The tube was let overnight at 4°C. Then, the ethanol was discarded after centrifugation at 2,500 × *g* and 4°C for 5 min. To carry out the cytometry analysis, the cells were washed with 1 ml of sodium citrate 38 mM pH 7.4. Then, the sodium citrate was discarded after centrifuging at 2,500 × *g* and 4°C for 5 min. Finally, the cells were resuspended in 500 μl of a solution containing sodium citrate 38 mM pH 7.4, 50 μg/ml of propidium iodide, and 5 μg/ml of RNase A (Sigma-Aldrich Co., St Louis, MO, United States) and incubated at 37°C for 20 min. After the addition of propidium iodide, the tubes were maintained permanently in the darkness until the cytometry lecture. The flow cytometry was performed in a flow cytometer Beckman Coulter Epics XL (Beckman Coulter S.A, Madrid, Spain). The cell populations corresponding to the different phases of the cell cycle (sub-G0, G0/G1, S and G2/M) were defined through their DNA quantity (Figure 9). The results were analyzed with the software FlowJo (FlowJo, United States).

### Immunoblot or Western Blot Studies

For the immunoblot studies, 325,000 PANC-1 cells were seeded into 6-cm-diameter plates and incubated at 37°C for 24 h. Then, the medium was changed and replaced with new medium containing TGM4 at different concentrations.

At the desired time of treatment, the medium was discarded and the plates were washed two times with cold PBS 1× and frozen at −80°C. Then, 300 μl of lysis buffer (20 mM HEPES, 2 mM EDTA, 0.5 mM EGTA, 1.5 mM MgCl2, 1 mM cantharidine, 1 mM orthovanadate) was added to the plate, and after 5 min, cells were scrapped and sonicated on ice.

The protein concentration determination of each sample was performed with the protein quantification DCTM kit (Bio-Rad, Barcelona, Spain), a colorimetric assay based on the Lowry method (Lowry et al., 1951).

The western blot was based on the work by Towbin et al. (1979). Samples were mixed with loading buffer (12 mM Tris–HCl pH 6.8, β-mercaptoethanol, 1%, SDS 0.2%, bromophenol blue 0.01%, and glycerol 50%) in a 1:10 proportion and boiled at 95°C for 5 min. Then, 30 μg of whole-cell lysate of each sample was loaded on a 10% SDS polyacrylamide gel. As a protein ladder standard, 2.5–5 μl of Precision Plus Protein all blue standard (Bio-Rad) was loaded.

The stacking gel was composed of 4% acrylamide–bisacrylamide, 166 mM Tris–HCl pH 6.8, 0.1% SDS, 1% ammonium persulfate, and 0.1% N,N,N′,N′-tetramethylethylenediamine. The separation gel was composed of 9.5% acrylamide–bisacrylamide, 1 M Tris-base pH 8.8, 0.1% SDS, 0.4% ammonium persulfate, and 0.04% N,N,N′,N′-tetramethylethylenediamine.

After loading all the samples in the SDS-polyacrylamide gels, the electrophoresis was set at 90 V for the stacking phase and changed to 120–140 V for the running phase. The electrophoresis buffer was composed of 19.2 mM Tris-base, 0.19 M glycine pH 8.6, and 0.1% SDS. When the electrophoresis was finished, the proteins into the gel were transferred to a nitrocellulose membrane (GE Healthcare, Kent, United Kingdom). The transfer process was performed in cold conditions and applying a constant amperage of 350–400 mA for 2 h with buffer consisting of 19.4 mM Tris-base, 0.19 M glycine, and 20% ethanol.

After finishing the transfer, the membrane was blocked in 5% skim milk in TBS (50 mM Tris–HCl, pH 7.6; 150 mM NaCl) for 30 min and then incubated in primary antibodies at 1:1,000 dilution with the exception of α-tubulin at 1:10,000 dilution. Primary antibody solution was prepared which contains 5% BSA and 0.1% Tween 20 (Sigma-Aldrich Co., St. Louis, MO, United States). The set of primary and secondary antibodies used were the following: antihuman rabbit polyclonal PARP (Santa Cruz Biotechnology, Inc., United States, #sc7150); antihuman mouse monoclonal CHOP (Cell Signaling Technology, Inc., United States, #2895); antihuman rabbit polyclonal BIP (Cell Signaling Technology, Inc., United States, #3177); antihuman rabbit polyclonal LC3B (Cell Signaling Technology, Inc., United States, #2775); antihuman mouse monoclonal DHFR (BD Transduction Laboratories, United States, #610697); antihuman mouse monoclonal Akt 1/2/3 (Santa Cruz Biotechnology, Inc., United States, #sc81434); antihuman rabbit polyclonal p-Akt (S473) (Cell Signaling Technology, Inc., United States, #4060); antihuman mouse monoclonal Jun (BD-Biosciences, United States, #J31920); antihuman rabbit polyclonal p-c-Jun (S63) (Cell Signaling Technology, Inc., United States, #2361); and antihuman mouse monoclonal α-tubulin (Sigma-Aldrich, United States, #T9026).

Membranes were incubated overnight at 4°C and washed two times for 5 min with TBS and Tween 20 0.1%. The membrane was incubated for 1 h in the dark with the secondary antibody (dilution 1:5,000) conjugated with a fluorochrome [IRDye 800CW Donkey Anti-Mouse IgG (H + L) or IRDye 800CW Donkey anti Rabbit IgG (H + L), LI-COR Biosciences, United States]. The secondary antibody was prepared in 2.5% skim milk in TBS with 0.1% Tween 20. Then, the secondary antibody was removed and the membrane was washed two times for 5 min in TBS and 0.1% Tween 20. The membranes were scanned in near-infrared spectroscopy (Odyssey Infrared Imaging System, LI-COR, Inc., Lincoln, NE, United States) with a resolution of 84 μM and analyzed with Image Studio™ software (LI-COR, Inc., Lincoln, NE, United States) obtaining the values of integrated optical density of each band. The α-tubulin content in each sample was used as a loading control.

### Confocal Microscopy

A tissue culture coverslip (Sarstedt, Germany) was placed into wells of 24-well plates. In total, 25,000 PANC-1 cells were seeded in each well and allowed to grow for 24 h. Then, the cells were transfected with the plasmid ptfLC3 (Kimura et al., 2007) (following Lipofectamine instructions), and after 24 h, TGM4 (35 μM) was added to the medium for 48 h. Cells were fixed with 4% (w/v) paraformaldehyde (Sigma-Aldrich, St. Louis; MO, United States) in PBS for 15 min and washed with PBS. Cells were permeabilized using Triton X-100 0, 1% for 10 min and washed with PBS. Finally, to avoid background usually due to autofluorescence, cells were incubated for 10 min with NH_4_Cl 100 mM. The coverslips with the fixed cells were stained with DAPI (Sigma-Aldrich, St. Louis, MO. United States) (1:500 in PBS for 5 min in darkness). Slides and coverslips were mounted together using VECTASHIELD HardSet Antifade Mounting Medium (Vector Laboratories, United States), allowing them to dry for 24 h in darkness and then storing the slides at 4°C and darkness until the analysis. Slides were observed under a confocal microscopy at 63× augments (Leica Microsystems, Wetzlar, Germany), and different pictures were collected whereas the samples were excited by the appropriate laser at the determined wavelength to be analyzed. In this experiment, cells were transfected with ptfLC3. This plasmid contains both red and green fluorescent proteins (RFP and GFP) tagged to the light chain 3 (LC3) protein. In autophagosomes, there will be yellow signals (overlap of red and green fluorescence) whereas autolysosomes appear with red fluorescent LC3 due to degradation of the GFP.

### Peroxisome Proliferator-Activated Receptor Gamma Activity

In total, 50,000 PANC-1 cells were seeded in 12-well plates. UAS-E1BTATA-LUC reporter and pRL-SV40P Renilla plasmids were cotransfected with the plasmid encoding the ligand-binding domain of peroxisome proliferator-activated receptor gamma (PPAR-γ) (Gal4-PPARGLB) following lipofectamine 2000 instructions (Park et al., 2003). After 24 h of transfection, cells were treated for 48 h with TGM4 at doses of 15, 25, and 35 μM. Rosiglitazone was used as positive control. Then, cells were subjected to dual-luciferase assay (Promega) following manufacturer’s instructions. Renilla expression values that are used as an internal control (Sherf et al., 1996). PPAR-γ activity (first lecture)/Renilla level (second lecture) ratios were used to analyze the results.

### *In vivo* Studies: Human Pancreatic Carcinoma Cells Xenograft Mice Model

NUDE mice [Swiss Crl:NU (Ico)-Foxn1 nu; Charles River Laboratories, France], aged between 4 and 6 weeks and with approximately 25 g of weight, were used. These animals were maintained under sterile conditions, kept on plastic cages located in a sterile closet (EHRET, Labor_U_Pharmatechnik, Deutschland) with a constant temperature of 28°C. The cabinet was kept in a room with a 12-h light/12-h dark schedule and a relative humidity of 40–60%. All the work or manipulation of the animals was conducted in every moment under sterile conditions on BSL-2 flow cabinets.

To study the *in vivo* effect of TGM4, a xenotransplant model was used. The cell line Mia-PaCa-2 was expanded and washed with PBS, and the trypsinized cells were collected in DMEM medium. Viable cells were counted and centrifuged at 600 × *g* for 5 min and resuspended in DMEM medium without FBS, to avoid immune reactions. A total of 7.5 × 10^6^ cells were injected subcutaneously with a 25-G caliber needle, in a volume of 150 μl. One week after inoculation, the tumors had approximated size of 5 mm and the treatment began.

The tumors size was measured once a week with a digital caliper. The tumor volumes were calculated by the formula: volume (mm^3^) = (2W × L)/2, where W represents the width and L represents the length of the tumor.

Animals’ treatments: To investigate the *in vivo* efficacy of TGM4, the animals were divided into three groups: treated with TGM4, gemcitabine, and control animals. A total of eight animals (four males and four females) were used for each condition. The treated animals received daily 1,000 mg/kg of TGM4 by cannulation. The control animals received nothing or 100 mg/kg of gemcitabine two times a week. Gemcitabine is the standard of care for the treatment of pancreatic cancer, and it was administrated through intraperitoneal injection. Oral administration is preferred since molecules are subjected to the processes of digestion and absorption. However, gemcitabine was given intraperitoneally since oral bioavailability is only 10% due to low intestinal permeability and oral absorption (Thompson et al., 2019). The duration of the experiment was 43 days. After the treatments, the animals were euthanized by decapitation.

All the protocols and procedures were revised and approved by the Comité Institucional de Investigación Animal (Comisión de Bioética de la Universitat de les Illes Balears). Project authorization was granted on 2018 by the Institutional Committee for Animal Research of the Balearic Government under the protocol number CEEA 94/05/18 (2018/09/AEXP).

### Data Analysis

All data shown in the graphs correspond to the mean values ± standard error of the mean (SEM) of at least three independent *in vitro* experiments (each duplicated) or cellular experiments. For animal studies, there is indicated in the graphs the number of animals used (*N*). The statistical analysis was performed by the average *S*tudent’s *t*-test, configured as unpaired, two-tailed test, with confidence intervals of 95%. For the statistical analysis of the animal studies, the non-parametric Mann–Whitney *U* test was used. All statistical analyses were performed using GraphPad Prism 5.0 program. The differences between experimental groups were considered statistically significant at *p* < 0.5. The different significances were represented as follows: *p < 0.05, ^**^p < 0.01, ^***^p < 0.001.

## Results

### Tri-2-Hydroxyarachidonein Impairs Cell Proliferation and Viability of Human Pancreatic Carcinoma and Human Pancreatic Carcinoma of Ductal Cell Lines

**FIGURE 2:**
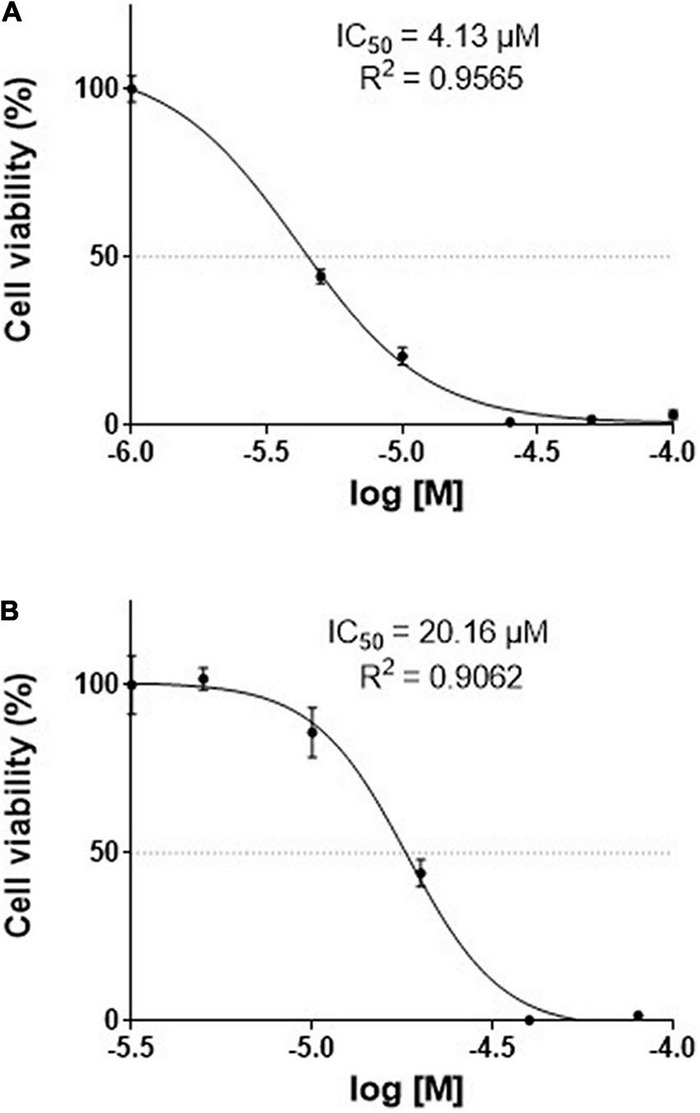
Effects of TGM4 on the proliferation of Mia-PaCa-2 **(A)** and PANC-1 **(B)** human pancreatic cell lines. Mia-PaCa-2 and PANC-1 were exposed to increasing doses (50–1000 μM) of TGM4 for 72 h, and cell viability was determined using the XTT method (*n* = 12). The IC_50_ values for TGM4 in Mia-PaCa-2 and PANC-1 were 20.16 and 4.13 μM, respectively.

Human pancreatic carcinoma and human pancreatic carcinoma of ductal cells were treated with different concentrations of TGM4, and cell death was measured by the XTT assay based on the mitochondrial function (succinate dehydrogenase activity). We observed that TGM4 (1–250 μM; 72 h) significantly impaired the growth of both PANC-1 (IC_50_ = 4.13 μM; *R*^2^ = 0.9565; Figure 2A) and Mia-PaCa-2 (IC_50_ = 20.16 μM; *R*^2^ = 0.9062; Figure 2B) human pancreatic cell lines.

### Tri-2-Hydroxyarachidonein Induces Cell Death in Human Pancreatic Carcinoma of Ductal Cells

**FIGURE 3:**
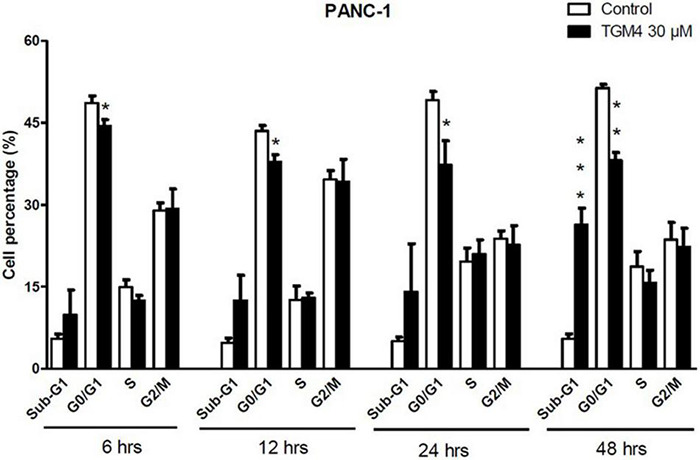
Tri-2-hydroxyarachidonein induction of sub-G1 and cell cycle arrest at G0/G1 of PANC-1 human pancreatic cells. Analysis of the DNA content (flow cytometry) of PANC-1 cells exposed to TGM4 (30 μM for 6, 12, 24, and 48 h) showing the proportion of cells in the cell cycle phases. Data represented as mean ± SEM (*n* = 3). Statistical significance was determined by unpaired two-tailed Student’s *t*-test (*p < 0.05; **p < 0.01; ***p < 0.001).

The proportion of cells in the different phases of the cell cycle was evaluated by measuring the intracellular DNA content after exposure to TGM4 (30 μM; 6, 12, 24, and 48 h). TGM4 treatment lowered the proportion of cells in the G0/G1 when compared to untreated controls as early as after 6 h of treatment (Figure 3) being the maximum observed after 48 h of treatment (control, 51.35 ± 0.65%; TGM4, 38.12 ± 1.42%, *p* < 0.01). On the other hand, after 48 h of treatment, TGM4 produced a significant increase in the sub-G0 values (control, 5.47 ± 0.90%; TGM4, 26.33 ± 3.05%, *p* < 0.001) when compared to untreated cells indicating DNA fragmentation and cell death. No significant differences in S and G2/M phases were observed during TGM4 exposure.

### Tri-2-Hydroxyarachidonein Induces Reticular Stress and Autophagy

**FIGURE 4:**
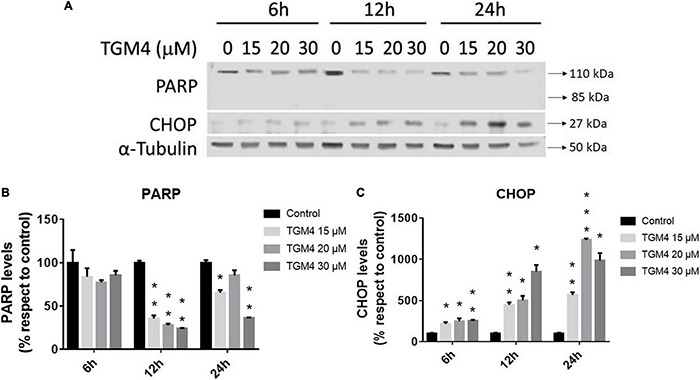
Protein expression analysis of PANC-1 cells treated with TGM4 at different doses and times. Representative immunoblots **(A)** and quantification **(B,C)** of PARP-1 and CHOP proteins. Data were normalized by α-tubulin. Each value represents the mean ± SEM (*n* = 3) expressed as a percentage with respect to control (100%). The significance was determined by a *t*-test (*p < 0.05; **p < 0.01; ***p < 0.001).

**FIGURE 5:**
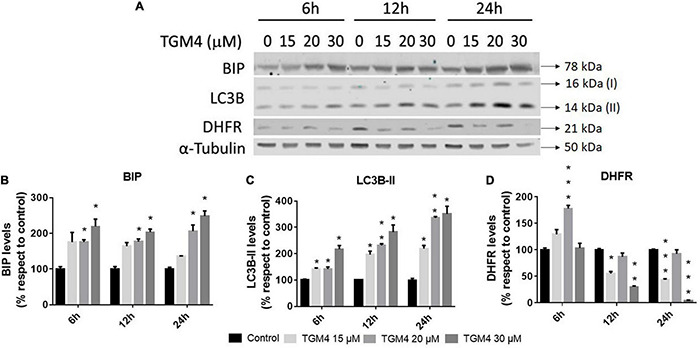
Protein analysis of PANC-1 cells treated with TGM4 at different doses and times. Representative immunoblots **(A)** and quantification **(B–D)** of BIP, LC3-II, and DHFR proteins. Data were normalized by α-tubulin. Each value represents the mean ± SEM (*n* = 3) expressed as a percentage with respect to control (100%). The significance was determined by a *t*-test (*p < 0.05; **p < 0.01; ***p < 0.001).

**FIGURE 6:**
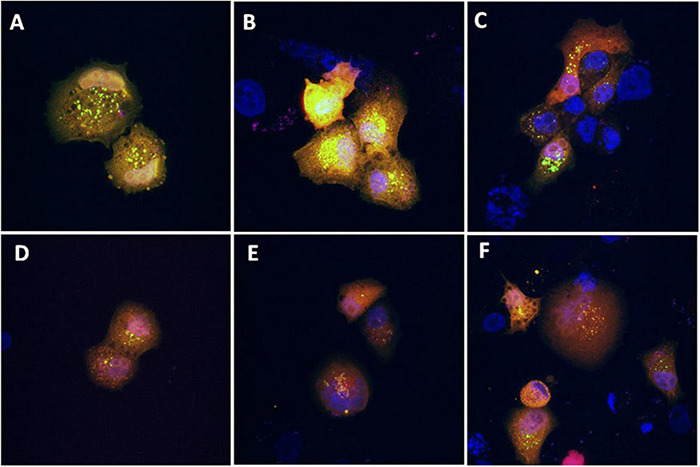
Evaluation of the effect of TGM4 on autophagic flux. Representative confocal microscopy images (630×) of PANC-1 cells transfected with the ptfLC3 (LC3-RFP-GFP) plasmid and incubated with vehicle **(A–C)** or TGM4 35 μM **(D–F)** for 48 h. Blue fluorescent signal (DAPI) marks the nucleus, whereas red and green signals represent LC3-B. After fusion of the autophagosome with lysosomes, green signal is degraded but not the red signal, due to differences in the isoelectric point between them.

As shown in Figure 3, the presence of the sub-G0 events after the exposure of PANC-1 cells to TGM4 indicates DNA damage and cell death. This feature was paralleled by the downregulation of PARP and upregulation of CHOP as shown in Figure 4. PARP is a 116 kDa nuclear polymerase, which can be cleaved by many caspases. Cleavage of PARP promotes cell death and is used as a marker of cells undergoing apoptosis (Oliver et al., 1998). CHOP is a protein which expression is induced by ER stress and is able to suppress cell cycle progression from G1 to S phase (Barone et al., 1994). PARP decreased after 12 h of treatment with 30 μM TGM4 from 100 ± 2.44% in controls to 23.92 ± 0.70% (*p* < 0.01), whereas CHOP was increased from 100 ± 8.70 to 850.24 ± 80.79% (*p* < 0.01). Although the fragmentation of PARP, a typical indicator of apoptosis, was not observed in our experiments, there was a loss of the full form of PARP as soon as 6 h after TGM4 treatment which also is indicative of cell death processes (Tempka et al., 2018). On the other hand, TGM4 treatment upregulated CHOP as soon after 6 h of treatment (Figure 4). CHOP upregulation initiates apoptotic cells (Hu et al., 2019). Since CHOP upregulation is marking endothelial reticulum-induced cell death, we next studied whether TGM4 induced the expression of BIP as well, a key marker of endothelial reticulum stress. Indeed, treatment of PANC-1 cells with TGM4 (30 μM) upregulated BIP expression (Figure 5). The protein levels of BIP were increased after 12 h of treatment from 100 ± 6.98% in control untreated cells to 203.32 ± 8.47% (*p* < 0.01). We next studied whether TGM4 treatment had activity in regulating other ER or autophagy markers as LC3B and DHFR. As can be seen in Figure 5, the bottom fragment of LC3B (LC3B-II) is upregulated as well after TGM4 treatment in a time- and dose-dependent manner. LC3B-II levels were increased after 12 h of treatment with 30 μM of TGM4 from 100 ± 1.48 to 282.12 ± 26.81% (*p* < 0.05) indicating an increase in autophagic flux. In this line of evidence, a representative example of the autophagic flux increase by TGM4 can be observed in Figure 6. Control PANC-1 cells (Figures 6A–C) showed more autophagosomes not fused with lysosomes (yellow and green signals) compared with PANC-1 cells treated with TGM4 (Figures 6B–D) were more autolysosomes (red signals; the fusion of the autophagosomes with lysosomes results in the degradation of GFP but not RFP producing red fluorescence) are observed. This result is further supported by the loss of DHFR (Figure 5). DHFR levels were decreased after 12 h of treatment with 30 μM of TGM4 from 100 ± 2.56 to 30.19 ± 1.57% (*p* < 0.01), which is also a marker of autophagic flux increase (Salvador et al., 2000; Majeski and Dice, 2004).

**FIGURE 7:**
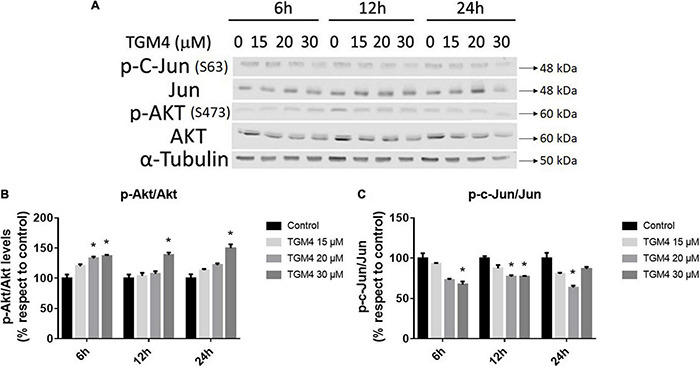
Protein analysis of PANC-1 cells treated with TGM4 at different doses and times. Representative immunoblots **(A)** and quantification **(B,C)** of p-AKT(S473)/AKT ratio and p-c-Jun(S63)/Jun ratio. Data were normalized by α-tubulin. Each value represents the mean ± SEM (*n* = 3) expressed as a percentage with respect to control (100%). The significance was determined by a *t*-test (*p < 0.05).

AKT and Jun are two oncogenes involved in proliferation and apoptosis and also mediate autophagy and tumorigenesis through the phosphorylation of a number of targets (Wang et al., 2012; Zhu et al., 2017). As observed in Figure 7, the ratio p-c-Jun/Jun decreased after 12 h of treatment with 30 μM of TGM4 from 100 ± 2.43 to 76.75 ± 1.17% (*p* < 0.05) whereas the ratio p-Akt/Akt increased after 12 h of treatment with 30 μM of TGM4 from 100 ± 6.103 to 138.59 ± 4.04% (*p* < 0.05).

### Tri-2-Hydroxyarachidonein Induces Peroxisome Proliferator-Activated Receptor Gamma Activation

**FIGURE 8:**
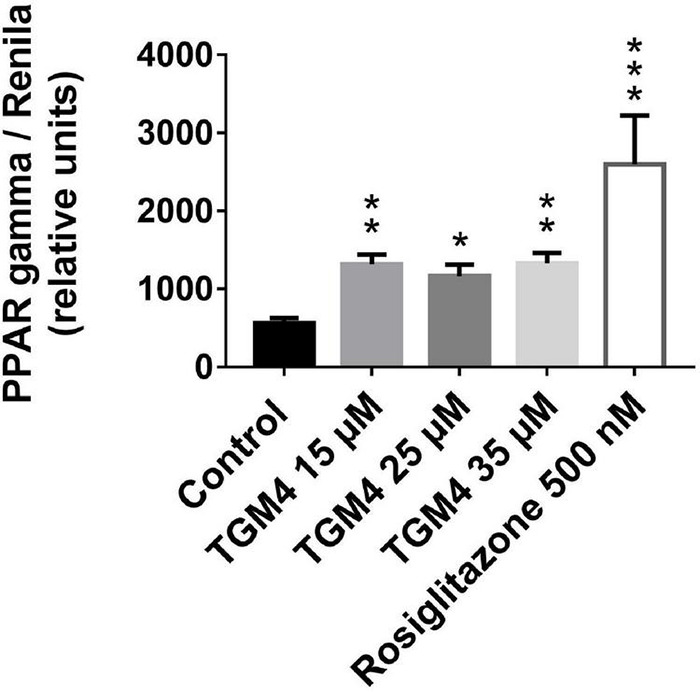
Effect of different doses of TGM4 on the activity of PPAR-γ on PANC-1 cells. PANC-1 cells were treated with 15, 25, and 35 μM of TGM4. PPAR-γ modulation was evaluated by the quantification of luciferase signal that is induced by PPAR-γ activity. Rosiglitazone (ROS) 500 nM was used as a positive control. The values were determined by luminescence measurement, and the mean ± SEM of 2 independent experiments with 3 replicates each expressed as a percentage with respect to the control were represented. The significance was determined by a *t*-test (*p < 0.05; **p < 0.01; ***p < 0.001).

It has been shown that arachidonic acid induces PPAR-γ activity (Kliewer et al., 1997), and in turn, PPAR-γ activation can induce autophagy (Soto-Avellaneda and Morrison, 2020). To further explore the cellular mechanism that explains the cytocidal autophagy induced by TGM4, we studied whether TGM4 was able to induce PPAR-γ activity. As is shown in Figure 8, TGM4 was able to induce the activity of PPAR-γ in the same range as rosiglitazone, a well-known PPAR-γ activator. PPAR-γ activity levels were increased from 595 ± 66.1 to 1251 ± 126.6 (*p* < 0.01); 1075 ± 151.7 (*p* < 0.05); 1311 ± 132.4 (*p* < 0.01) after 48 h of treatment with TGM4 at doses of 15, 25, and 35 μM, respectively.

### Tri-2-Hydroxyarachidonein Effect on the Progression of Human Pancreatic Carcinoma Cell Line Xenograft in Nude Immunodepressed Mice

**FIGURE 9:**
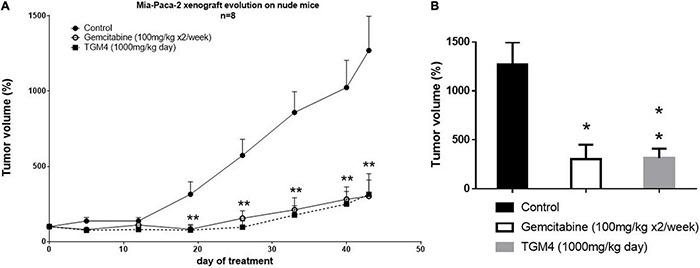
Effect of TGM4 in xenograft model of pancreatic cancer (Mia-PaCa-2). A total of 7.5 × 106 Mia-PaCa-2 were injected subcutaneously in immunosuppressed nude Swiss Crl: NU (Ico) – Foxn1nu mice. Control animals received nothing or subcutaneous injections of 100 mg/kg of gemcitabine two times a week. The treated animals received through cannulation 1,000 mg/Kg of TGM4. **(A)** Changes in the tumors volumes through 43 days of treatment represented as percentage respect day 0. Significance indicates TGM4 against control. **(B)** Percentage of tumors volumes at day 43 of treatment. Each value represents the average of the percentages ± SEM (*n* = 8) relative to day 0 (taken as 100%); non-parametric Mann–Whitney *U* test (*p < 0.05; **p < 0.01).

Finally, the TGM4 antitumor activity was tested *in vivo* on a xenograft mice model. The tumor was induced by subcutaneous injection of 7.5 × 106 Mia-PaCa-2 cells per mouse. TGM4 was administrated orally and daily at a dose of 1,000 mg/kg of body weight. Control mice received nothing or gemcitabine 100 mg/kg i.p. two times a week, the standard of care for the treatment of pancreatic cancer. The experiment lasted for 43 days, during which the tumor volume was measured. After 43 days of treatment, the average size of the tumors of non-treated animals was 1269.58 ± 227.74 mm^3^ whereas the average size of the tumors of animals treated with gemcitabine or TGM4 was 302.60 ± 148.36 and 314.57 ± 95.38, respectively (*p* < 0.001) (Figure 9).

## Discussion

An intricate network of signaling pathways governs pancreatic cancer progression (Polireddy and Chen, 2016). In fact, pancreatic cancer demonstrated hundreds of chromosomal structural variants per patient (Jones et al., 2008; Waddell et al., 2015). Unfortunately, because of this complex landscape, there are no effective therapies available to date, and novel conceptual approaches are needed. Among these novel approaches, the use of synthetic lipid molecules to interfere with the proliferative events of cancer cells is indeed worth to be investigated.

During the past decades, it has been demonstrated a lipid metabolic reprogramming in cancer cells (Fernández et al., 2020) and that the proportion of membrane lipid species is altered in a number of tumors including pancreatic cancer (Meng et al., 2004; Casares et al., 2019).

As classical examples of the role of lipid molecules in the cancer cell fate, it is well known that ceramides play a major role in the regulation of apoptosis (Riboni et al., 2002) and that glycosphingolipid species aberrantly expressed in tumors (Hettmer et al., 2005) induce angiogenesis (Birklé et al., 2003). In this conceptual frame, our group demonstrated that sphingomyelin synthase 1 and sphingomyelin synthase 2 showed opposite associations with the survival of patient with glioma (Fernández-García et al., 2019). Recently, we proved that the use of synthetic lipid molecules can indeed change the cell signaling events associated with a variety of cancers. In this regard, we demonstrated that the synthetic fatty acid 2-hydroxyoleic acid induced cytotoxic autophagy in glioma cells lines and in human glioma animal models (Marcilla-Etxenike et al., 2012; Teres et al., 2012; Beteta-Göbel et al., 2021). In fact, 2-hydroxyoleic acid is currently in a phase IIB/III randomized, double-blind, placebo-controlled trial in subjects with newly diagnosed primary glioblastoma multiforme (NCT04250922) and in an open-label, non-randomized study in pediatric patients with advanced high-grade gliomas and other solid tumors (NCT04299191).

Previous work of our group also showed that hydroxytriolein has an antitumor efficacy against non-small cell lung cancer (NSCLC). Hydroxytiolein is a synthetic 2-hydroxy fatty-acyl triolein derivative that induces the membrane translocation and activation of PKC and ERK, and also the production of reactive oxygen species and macroautophagy (Guardiola-Serrano et al., 2015).

Now, we investigated a synthetic lipid termed TGM4, based on the structure of the synthetic lipid 2-hydroxy-arachidonic acid with the aim to induce cytotoxic autophagy in pancreatic tumor cell lines. The rationale behind the synthesis of TGM4 is that arachidonic acid is able to induce autophagy in a variety of cell types (O’Rourke et al., 2013). Our *in vitro* studies clearly showed that TGM4 decreases the number of viable pancreatic tumor cells (Mia-PaCa-2 and PANC-1) in a time- and concentration-dependent manner (Figure 2). These results were paralleled by an increase of sub-G0 population events (Figure 3) indicating the induction of cell death by TGM4. In this regard, PARP expression and fragmentation were analyzed as an indicator of apoptosis. As shown in Figure 4, fragmentation of PARP was not observed after treatment with TGM4 but a downregulation of full-length PARP (116 kDa) which may mark cell death since downregulation of full-size PARP protein results also in DNA damage and decreased viability (Tempka et al., 2018). In addition, TGM4 treatment induced CHOP upregulation (Figure 4) a well-known marker and initiator of apoptotic cell death if endothelial reticulum homeostasis is not restored (Hu et al., 2019). CHOP is considered the link between endothelial reticulum stress and autophagy (B’chir et al., 2014). Under endothelial stress conditions, CHOP promotes IRE1α signaling pathway activation and autophagy (Shimodaira et al., 2014). Moreover, the upregulation of CHOP by TGM4 was paralleled by the upregulation of BIP (Figure 5), and one of the master regulators of the UPR/autophagy and represents a pharmaceutical target to induce autophagy and apoptosis in melanoma cancer cells (Cerezoa and Rocchib, 2017).

The aggregation and posttranscriptional modification of microtubule-associated protein LC3, a mammalian homolog of yeast Atg8, has been also used as a specific marker to monitor autophagy (Kuma et al., 2007). LC3B is first cleaved at the carboxy terminus immediately following synthesis to yield a cytosolic form LC3B-I. During autophagy, LC3B-I is converted to LC3B-II by a ubiquitin-like system involving Apg7 and Apg3 that allows for LC3 to become associated with autophagic vesicles (Ichimura et al., 2000; Kabeya et al., 2000; He et al., 2003; Tanida et al., 2004; Wu et al., 2006). As shown in Figure 5, TGM4 induced a marked overexpression of LC3B-II suggesting an increase in the autophagic flux (Figure 6) and consequently that autophagy is triggered after TGM4 treatment. In addition to the overexpression of LC3B-II, the use of the plasmid that expresses LC3B tagged with RFP and GFP allowed us to confirm, after treatment with TGM4, the fusion of the autophagosomes with the lysosomes and the consequent formation of autolysosomes. The low pH of the autolysosomes degrades the green signal while not altering red signal due to differences in the isoelectric point of both fluorescent proteins. In this line of evidence, the downregulation of DHFR (Figure 5) also argues in favor of autophagy and autophagosome activity since degradation of DHFR occurs in autophagosomes. In fact, DHFR has been used to investigate the uptake of cytosolic proteins into lysosomes for degradation by the chaperone-mediated autophagic pathway (Salvador et al., 2000; Majeski and Dice, 2004). Moreover, downregulation of DHFR by the synthetic fatty acid HOA hampers glioma and leukemia cancer cells replication (Lladó et al., 2009).

Among the lipids that induce autophagy, the role of ω-6 polyunsaturated fatty acids is remarkable (O’Rourke et al., 2013). In this regard, supplementation with arachidonic acid and di-homo-γ-linoleic acid leads to the increase of autophagic marker LC3-II in HeLa cells together with downregulation of p62 (O’Rourke et al., 2013). The formation of LC3 puncta in HeLa cells after treatment with arachidonic acid or DGLA is diminished by the inactivation of the autophagy gene ATG161L indicated the capacity of ω-6 PUFAs to activate the autophagic flux in HeLa cells (O’Rourke et al., 2013).

The cellular mechanisms that are responsible for the induction of cytocidal autophagy by TGM4 are unknown; however, our results on PPAR-γ activation (Figure 8) suggest at least a partial role of the peroxisome proliferator-activating factors. These nuclear receptors which are activated by lipid signals control the expression of autophagy and autophagy-related genes (Soto-Avellaneda and Morrison, 2020 and references therein).

Tri-2-hydroxyarachidonein has demonstrated to interfere with the PI3K/AKT and Jun pathways. As shown in Figure 7, TGM4 induced the upregulation of p-AKT and downregulation of p-c-Jun as soon as 6 h after treatment. We hypothesize that these effects could be a part of a secondary survival cellular mechanism to counteract the activation of the endothelial reticulum and cell death pathways since Akt inhibition promotes autophagy (Degtyarev et al., 2008) and activation of JNK reduces autophagy and increases apoptosis providing a promising strategy for prostate cancer therapy (Zhu et al., 2017).

Finally, to validate the effects of TGM4 in organic tumors, we used a nude immunodepressed mice model. As shown in Figure 9, the effect of the TGM4 at a daily oral dose of 1,000 mg/kg is very similar to that produced by the drug of reference gemcitabine at a dose of 100 mg/kg given to the mice by intraperitoneal injection two times a week.

We demonstrated that TGM4 induces pancreatic cell death by the activation of autophagy and related signal transduction events in cell cultures and reduces tumor growth in an *in vivo* xenograft model. This work highlights the importance of lipid signaling in cancer and the use of synthetic lipid structures, as TGM4, as a novel approach to treat pancreatic cancer and other neoplasias.

## Data Availability Statement

The raw data supporting the conclusions of this article will be made available by the authors, without undue reservation.

## Ethics Statement

The animal study was reviewed and approved by the Comité Institucional de Investigación Animal (Comisión de Bioética de la Universitat de les Illes Balears).

## Author Contributions

JF-D, RB-G, MT, JC, PF-G, VL, PE, and XB discussed the concepts, wrote the parts, and reviewed the entire manuscript. All authors contributed to the article and approved the submitted version.

## Conflict of Interest

PF-G and VL were employed by company Laminar Pharmaceuticals. The remaining authors declare that the research was conducted in the absence of any commercial or financial relationships that could be construed as a potential conflict of interest.

## Publisher’s Note

All claims expressed in this article are solely those of the authors and do not necessarily represent those of their affiliated organizations, or those of the publisher, the editors and the reviewers. Any product that may be evaluated in this article, or claim that may be made by its manufacturer, is not guaranteed or endorsed by the publisher.
